# Endophytic fungi from kale (*Brassica oleracea* var. *acephala*) modify roots-glucosinolate profile and promote plant growth in cultivated *Brassica* species. First description of *Pyrenophora gallaeciana*

**DOI:** 10.3389/fmicb.2022.981507

**Published:** 2022-10-05

**Authors:** Jorge Poveda, Víctor M. Rodríguez, María Díaz-Urbano, František Sklenář, Zaki Saati-Santamaría, Esther Menéndez, Pablo Velasco

**Affiliations:** ^1^Institute for Multidisciplinary Research in Applied Biology (IMAB), Universidad Pública de Navarra, Pamplona, Spain; ^2^Group of Genetics, Breeding and Biochemistry of Brassicas, Mision Biologica de Galicia (MBG-CSIC), Pontevedra, Spain; ^3^Laboratory of Fungal Genetics and Metabolism, Institute of Microbiology of the Czech Academy of Sciences, Prague, Czechia; ^4^Microbiology and Genetics Department and Institute for Agribiotechnology Research (CIALE), University of Salamanca, Salamanca, Spain

**Keywords:** sinigrin, *Fusarium oxysporum*, *Setophoma terrestris*, *Acrocalymma vagum*, *Brassica* U’s triangle, indoleacetic acid, glucosinolates

## Abstract

Endophytic fungi of crops can promote plant growth through various mechanisms of action (i.e., improve nutrient uptake and nutrient use efficiency, and produce and modulate plant hormones). The genus *Brassica* includes important horticultural crops, which have been little studied in their interaction with endophytic fungi. Previously, four endophytic fungi were isolated from kale roots (*Brassica oleracea* var. *acephala*), with different benefits for their host, including plant growth promotion, cold tolerance, and induction of resistance to pathogens (*Xanthomonas campestris*) and pests (*Mamestra brassicae*). In the present work, the molecular and morphological identification of the four different isolates were carried out, describing them as the species *Acrocalymma vagum*, *Setophoma terrestris, Fusarium oxysporum*, and the new species *Pyrenophora gallaeciana*. In addition, using a representative crop of each *Brassica* U’s triangle species and various *in vitro* biochemical tests, the ability of these fungi to promote plant growth was described. In this sense, the four fungi used promoted the growth of *B. rapa*, *B. napus*, *B. nigra, B. juncea,* and *B. carinata*, possibly due to the production of auxins, siderophores, P solubilization or cellulase, xylanase or amylase activity. Finally, the differences in root colonization between the four endophytic fungi and two pathogens (*Leptosphaeria maculans* and *Sclerotinia sclerotiorum*) and the root glucosinolate profile were studied, at different times. In this way, how the presence of progoitrin in the roots reduces their colonization by endophytic and pathogenic fungi was determined, while the possible hydrolysis of sinigrin to fungicidal products controls the colonization of endophytic fungi, but not of pathogens.

## Introduction

Endophytic fungi live asymptomatically inside plant tissues. In agriculture, endophytic fungi have been isolated from roots and aerial parts of several crops, mostly cereals (barley, maize, rice, sugarcane, and wheat) and soybean (Rana et al., 2019). These fungi have important benefits for their host plants by increasing tolerance to abiotic stresses (Khan et al., 2015) and resistance to biotic stresses, through direct action or by activating plant defensive responses (Poveda et al., 2020a; Poveda and Baptista, 2021). Furthermore, endophytic fungi promote plant growth by increasing access to nutrients (nitrogen, phosphorus, potassium, iron, zinc, etc.), production of plant hormones, reduction of the plant ethylene concentration, or an increased water acquisition rate (Poveda et al., 2021a). However, the use of endophytic fungi in a directed way in agriculture requires knowledge of their biological capacities and their behaviors in different crops and environments, the development of a large-scale production system, and their release to the market (Murphy et al., 2018). In that sense, in recent years numerous patents have been filed (i.e., 185 patents between 1988 and 2019 [Al-Ani, 2019; Ortega et al., 2020]), on the use of isolates from genera such as *Neotyphodium*, *Muscodor*, *Curvularia,* and *Fusarium.*

Plant species within the genus *Brassica* include important crops both agronomically and economically worldwide (Francisco et al., 2017). The U’s triangle classically explains the basic systematic relationship between the six major *Brassica* species (Nagaharu and Nagaharu, 1935). This system proposes that the three tetraploid species *B. juncea* (AABB genome), *B. napus* (AACC), and *B. carinata* (BBCC) are the derived allotetraploids of the diploid species *B. rapa* (AA), *B. nigra* (BB), and *B. oleracea* (CC), respectively, which arose by natural hybridization and chromosome doubling (Kim et al., 2018). Among the diploid species, kale (*B. oleracea* var. *acephala*) is a crop characterized by the formation of leaves along the stem, which are popular nowadays as a “superfood” as they contain phytochemicals beneficial to people’s health (Šamec et al., 2020).

*Brassica*s represent a unique case for us to study due to the production of glucosinolates (GSLs). GSLs are a class of sulfur-containing compounds found predominantly in the Brassicaceae plant family (Wu et al., 2021). These secondary metabolites are involved in numerous plant physiological processes, including adaptation to environmental conditions (Poveda et al., 2021b) and defense against pests and diseases (Eugui et al., 2022), including pathogenic fungi (Poveda et al., 2020b). Therefore, pathogenic fungi need to develop tolerance mechanisms to GSLs that infect *Brassica* tissues (Vela-Corcía et al., 2019). In this sense, endophytic fungi from horseradish (*Armoracia rusticana*) (Brassicaceae family) have been described as colonizing the roots when GSLs are used as a nutrient resource (Szűcs et al., 2018).

Conversely, few studies have focused on the diversity of endophytic fungi and their role as plant stimulators in *Brassica* crops (Card et al., 2015; Poveda et al., 2022). A previous study described, for the first time, the diversity of root endophytic fungi in kale roots, and how some isolates could promote plant growth and resilience (Poveda et al., 2020c).

Against that background, the objectives of this work were to study the biological properties of the four endophytic fungi widely isolated in kale and determine the role of GSLs in the colonization of these fungi using the *Brassica* U’s triangle.

## Materials and methods

### Biological material

The endophytic fungi used were isolated from *B. oleracea* var. *acephala* (kale) and identified by sequencing the internal transcribed spacer 1 (ITS1), 5.8S rDNA, and ITS2 as *Fusarium* sp. (MT628384), Pleosporales sp. A (MT628351), Pleosporales sp. B (MT628399), and *Acrocalymma* sp. (MT626728) (Vela-Corcía et al., 2019).

To test the role of GSLs in root colonization, two brassica root pathogenic fungi were used, *Sclerotinia sclerotiorum* and *Leptosphaeria maculans.* The *S. sclerotiorum* isolate MBG-Ss2 was originally isolated from naturally infected *B. napus* in an experimental field at MBG in January 2008 (Madloo et al., 2019). The *L. maculans* isolate CRD13/125/99 was provided by the Regional Diagnostic Center of the Regional Government of Castile and Leon (Salamanca, Spain), having been isolated from a *B. napus* field in Palencia (Spain) (Poveda, 2022).

The local kale (*B. oleracea* var. *acephala*) variety MBG-BRS0106 was utilized for *in planta* assays. Furthermore, for the growth promotion study of *Brassica* U’s triangle crops, *B. oleracea* (MBG-BRS0462), *B. napus* (MBG-BRS0063), *B. rapa* (MBG-BRS0163), *B. juncea* (EXT-BRS0184), *B. nigra* (EXT-BRS0185), and *B. carinata* (EXT-BRS0219) were used.

### Taxonomic study of endophytic fungi

#### DNA extraction, amplification, and sequencing

Fungal DNA was extracted from mycelium scraped from a PDA culture using a commercial kit (Phire Plant Direct PCR Kit, Thermo Fisher Scientific). A ribosomal DNA region including the internal transcribed spacer 1 (ITS1), 5.8S rDNA, and ITS2 was amplified by PCR using primers ITS1 and ITS4 (White et al., 1990). The glyceraldehyde-3-phosphate dehydrogenase (GADPH) gene was amplified using the primer pair gpd-1 and gpd-2 (Berbee et al., 1999). The RNA polymerase II gene was amplified using RPB2-5 and RPB2-7c, and RPB2-7 and RPB2-11a (Liu et al., 1999). For large subunit rDNA (LSU), the LROR and LR5 primer pair was used (Vilgalys and Hester, 1990). Amplification conditions were 5 min at 98°C, 35 cycles at 98°C for 30 s, 54°C for 30 s, and 72°C for 60 s. PCR amplicons were purified following the manufacturer’s instructions (MSB Spin PCRapace, Stratec Biomedical, Germany) and sequenced through the Genomics Service at CACTI, University of Vigo, Spain.^1^ The GenBank accession numbers can be found in Supplementary Table S1.

#### DNA sequence analyses

The sequences obtained using the forward and reverse primers were assembled using the software package Seqman (v. 10.0.1) from DNAStar Inc. The resulting sequences were used for a BLAST search against the GenBank database. The datasets for phylogenetic analysis were obtained by downloading the sequences of accepted, closely related species from GenBank. Multiple sequence alignments were created in MAFFT v.7 (Katoh and Standley, 2013) using the G-INS-i method and edited in AliView v. 1.27 (Larsson, 2014). A suitable model of evolution for each alignment was selected in jModelTest v. 2.1.7 (Posada, 2008) using the BIC (Bayesian information criterion). The phylogenetic position of strains H22, H64, and H890 was demonstrated through maximum likelihood analysis of ITS (rDNA) in IQ-TREE v. 2.1.2 (Minh et al., 2020). The statistical support was determined by 1,000 standard bootstrap replicates. In the case of isolate H441, which is described as a new species in this study, single-locus maximum likelihood analysis was performed in the same way as mentioned above using DNA sequences of four loci, ITS (rDNA), LSU (rDNA), GAPDH, and RPB2. Furthermore, concatenated phylogenetic trees were calculated using partitioned analysis IQ-TREE v. 2.1.2 based on sequences of all four loci. The resulting phylogenetic trees were prepared in iTOL (Interactive Tree Of Life) (Letunic and Bork, 2021). Only significant bootstrap values (higher than 70) are presented in the final phylograms.

#### Fungal morphological study

Fungi were cultivated on potato dextrose agar (PDA) (Sigma-Aldrich, St. Louis, United States) at 25°C in a 12 h day/night cycle for 5 days. Morphologies of fungi structures were investigated using a Leica DM4500 microscope (Leica, Germany) equipped with a Retiga 2000R camera (QUIMAGING, Canada). At least 24 measurements per structure were taken.

### *In vitro* plant growth promoting assays

Solubilization of insoluble phosphate (CaH_2_PO_4_) and potassium (AlKO_6_Si_2_) was performed using YED-P and Aleksandrov media, respectively (Hu et al., 2006; Jiménez-Gómez et al., 2020). If a clear halo around the mycelium appeared within 7 days of incubation at 28°C, it was considered a positive result. Auxins and siderophore production were analyzed by the colorimetric method (Khalid et al., 2004) and in M9-CAS-agar medium (Jiménez-Gómez et al., 2019), respectively. The production of siderophores was analyzed qualitatively, by means of the production of specific yellow halos. For the quantification of auxins produced, a standard line was made with the 3-indoleacetic acid (IAA) standard (I3750, Sigma-Aldrich, Missouri United States) by spectrophotometry at 530 nm.

### Enzymatic activity assays

To detect cellulolytic, xylanolytic, or amylolytic activities, TSA medium was supplemented with 1% CMC (carboxymethyl cellulose), xylan, or starch, respectively (Garcia-Fraile et al., 2007). Briefly, a mycelium plug of each fungal isolate was placed on a different screening plate, which were incubated at 24°C for 7 days. Appearance of halos, which were observed after staining, indicated the degradation of the substrate. Cellulose- and xylan-supplemented plates were soaked in Congo Red and clarified with 1 M NaCl solution (Mateos et al., 1992). Starch-supplemented plates were soaked with lugol solution (potassium iodine, Panreac®) for a few seconds, until a halo appeared (Petzel and Hartman, 1986).

### *In planta* growth promotion In *Brassica* U’s triangle crops

For the *in planta* assays, a representative of each of the vertices and edges of the *Brassica* U’s triangle was used: *B. napus*, *B. rapa*, *B. oleracea* var. *acephala*, *B. juncea*, *B. nigra,* and *B. carinata*. The plants were grown in 10 l pots with sterile (80°C, 24 h) peat substrate (Profi-Substract, Gramoflor, Valencia, Spain) and their roots inoculated with each endophytic fungus. Twelve plants were inoculated per fungus, leaving 12 plants uninoculated as a control. Fungal inoculation was performed as described by Poveda et al. (2019) for *B. napus*. At 4 weeks post-fungi inoculation, fresh and dry (48 h at 60°C) weights were taken from the aerial parts of the plants.

### Root colonization assays with endophytic and pathogenic fungi

Following the methodology described in the previous section, kale plants were inoculated with the four endophytic fungi and two pathogenic fungi, to analyze colonization differences related to the profile of root GSLs for different modes of interaction with *Brassica* plants. The plants were grown in 3 l pots with a substrate consisting of peat moss (Profi-Substract, Gramoflor, Valencia, Spain) previously treated at 80°C for 24 h, and their roots were inoculated with each fungus. Thirty 3-week-old plants were inoculated per fungus with 1 ml of a conidial suspension at 2 × 10^7^ spore ml^−1^ (determined using a hemocytometer), keeping a further 30 plants free of inoculation as controls. For *S. sclerotiorum,* all the mycelium formed in three PDA Petri dishes was collected and mixed with 30 ml of sterile distilled water in a Falcon tube. Subsequently, 1 g of Ballotini glass balls (0.15–0.25 mm and 1 mm diameter; 0.5 + 0.5 g, respectively) (Potters, Saint-Pourçain-sur-Sioule, France) was added, vigorously shaking for 20 min. A mycelium suspension was obtained, which was adjusted to the absorbance of 0.17 per mL at 520 nm, for inoculation.

Subsequently, the roots of nine plants per fungal inoculation were collected and washed with water (freezing them directly in liquid nitrogen; three pools, each representing all the roots of three different plant), at different post-fungi-inoculation times: 7, 12, and 17 days. The different root pools were used to quantify the fungal root colonization by qPCR and GSL profiles.

#### Quantification of fungal root colonization

Quantification of fungal DNA in kale roots was performed by qPCR as previously described by Velasco et al. (2021), with some modifications. DNA was extracted from roots of the untreated (control) and fungal (endophytes and pathogens)-inoculated plants, using the methodology previously described in this work (section 2.2.1). A mix was prepared in a 20 μl volume using 100 ng of DNA, 10 μl of Brilliant SYBR Green QPCR Master Mix (Roche, Penzberg, Germany), the forward and reverse primers at a final concentration of 600 nM, and nuclease-free PCR-grade water (to adjust the final volume). Different endogenous genes of each of the fungi and kale were used (Supplementary Table S2). Amplifications were performed in a 7,500 Real-Time PCR System (Applied Biosystem, Forster City, CA, United States) programmed for 40 cycles under the following conditions: denaturation, 95°C for 15 s; annealing, 60°C for 1 min; extension, 72°C for 1 min. DNA extracted from the root pools of each fungal inoculation and for each post-inoculation time was used, performing each PCR in triplicate. Cycle threshold values assisted us to calculate the amount of fungal DNA using standard curves. The values obtained for fungus DNA were referenced against the values obtained for kale DNA in each of the samples.

#### GSLs analysis

Analysis of the GSL profiles in the samples was carried out following the methodology described by Kliebenstein et al. (2001), with minor modifications. Twelve milligrams of freeze-dried kale root powder was mixed with 400 μl of 70% (v/v) methanol preheated to 70°C, 10 μl of PbAc (0.3 M) and 120 μl of ultra-pure water preheated to 70°C, and 20 μl of glucotropaeolin (added as the internal standard). After shaking in a microplate incubator (OVAN Orbital Midi; OVAN, Badalona, Spain), 400 μl of the glucosinolate extracts were pipetted into an ion-exchange column with Sephadex DEAE-A25 (Sigma-Aldrich, St. Louis, MO, United States). For desulfation, purified sulfatase (E.C. 3.1.6.1, type H-1 from *Helix pomatia*; Sigma-Aldrich, St. Louis, MO, United States) was added. Finally, the desulfated GSLs were diluted in 200 μl of ultra-pure water and 200 μl of 70% methanol and kept frozen for further analyses.

The chromatographic analyses were conducted on a UHPLC (Nexera LC-30 AD; Shimadzu, Kyoto, Japan) equipped with an injector (Nexera SIL-30 AC, Shimadzu, Kyoto, Japan) and one SPDM20A UV/VIS photodiode array detector (Shimadzu, Kyoto, Japan). UHPLC column X Select ®HSS T3 (2.5 μm, 2.1 × 100 mm i.d.) from Waters (Waters Corporation, Milford, MA, United States) was used, which was protected with a VanGuard pre-column. GSLs were separated in aqueous acetonitrile, with a flow of 0.5 ml min^− 1:1.5^ at 100% H_2_O, an 11 min gradient from 5 to 25% (v/v) acetonitrile, 1.5 min at 25% (v / v) acetonitrile, a minute gradient from 25 to 0% (v/v) acetonitrile, and a final 3 min at 100% H_2_O; then, they were quantified at 229 nm. Identification of the GSLs was carried out by comparison with commercial standards (Phytoplan, Heidelberg, Germany).

### Statistical analysis

To compare the means for each fungal inoculation treatment with the control, Student’s *t*-test was used at *p* < 0.05 for each assay, using the measurements of 10 plants per treatment for plant growth quantification and the measurements of three root pools with three complete roots each for GSLs analysis. To quantify the colonization, one-way ANOVA using Tukey’s multiple range test was used, by analyzing the measurements of three root pools with three complete roots each.

## Results

### Phylogenetic analysis

**Figure 1:**
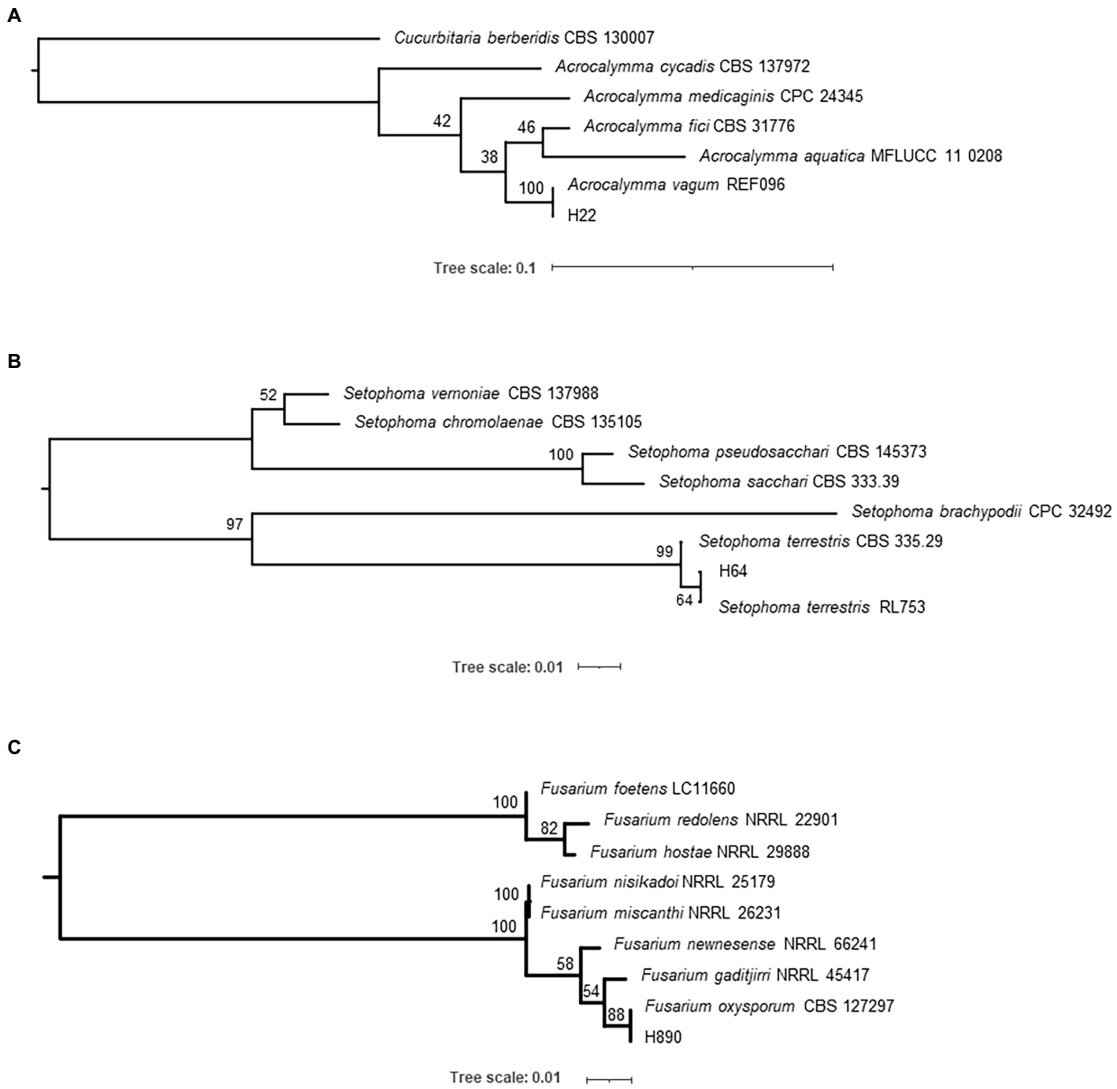
Phylogenetic trees displaying the relationships between isolates H22 **(A)**, H64 **(B)**, and H890 **(C)**. The trees were calculated in IQ-TREE 2.1.2 using ITS (rDNA) sequences with a TrNef+G substitution model and 1,000 standard bootstrap repeats.

**Figure 2:**
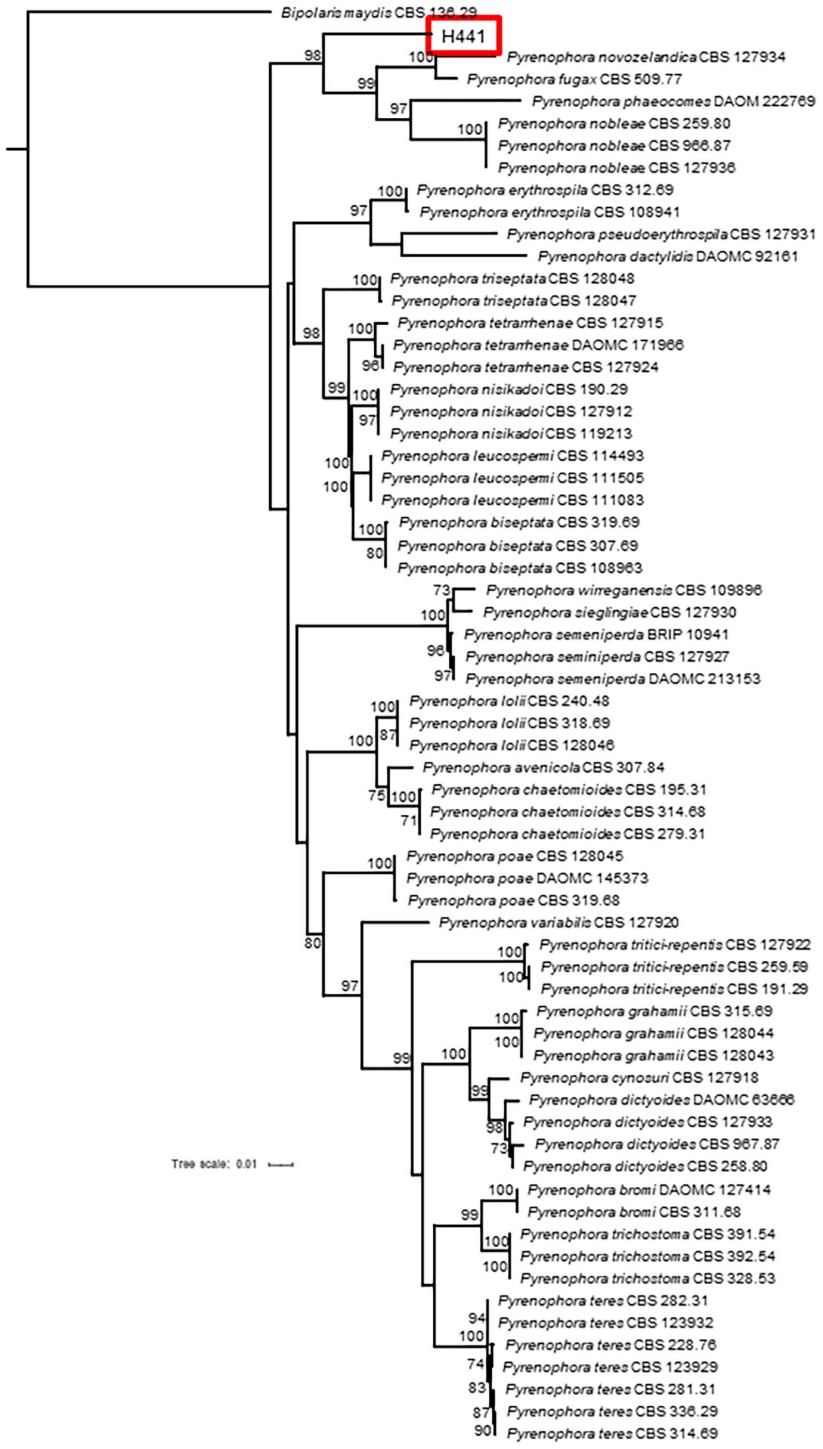
Phylogenetic tree displaying the relationship of isolate H441 with several species of genus *Pyrenophora*. The tree was calculated by partitioned analysis in IQ-TREE 2.1.2 using DNA sequences from four loci (GAPDH—HKY + I + G; ITS—TrNef+I + G; LSU—K80 + I; RPB2—K80 + I + G) and 1,000 standard bootstrap repeats.

To identify the four isolates, several phylogenetic trees were created. Identification of the fungal strains isolated in this study was carried out through a BLAST search against the GenBank database using ITS sequences, which gave the following results: strain H22 (formerly named: *Acrocalymma* sp.; Poveda et al., 2020a) is a representative of *Acrocalymma vagum*, H64 (formerly named: Pleosporales sp. A; Poveda et al., 2020a) of *Setophoma terrestris*, and H890 (formerly named: *Fusarium* sp.; Poveda et al., 2020a) of *Fusarium oxysporum*. The results of our maximum likelihood analysis representing the phylogenetic relationships with closely related species are depicted in Figure 1 and the fungal morphology in Supplementary Figures S1–S3. On the other hand, the BLAST search for strain H441 (formerly named: Pleosporales sp. B) failed to identify the strain to the species level. Based on the results of a phylogenetic analysis using the four DNA loci depicted in Figure 2 and Supplementary Figure S4, strain H441 was described as the new species *Pyrenophora gallaeciana* sp. nov., forming a clade with *P. nobleae*, *P. fugax*, *P. novozelandica*, and *P. phaeocomes* in single-locus trees based on ITS and GAPDH, and clustering with *P. chaetomioides* and *P. lolii* in an RPB2 tree (albeit with very low support). The tree based on LSU sequences was poorly resolved because of little variation in the alignment. In the tree calculated from all four loci, *P. gallaeciana* was located in the clade with *P. nobleae*, *P. fugax*, *P. novozelandica*, and *P. phaeocomes*. This clade, as well as other deep nodes, acquired quite high bootstrap support, unlike trees based on single-locus alignment, but this is likely an artifact caused by concatenation (Kubatko and Degnan, 2007).

**Figure 3:**
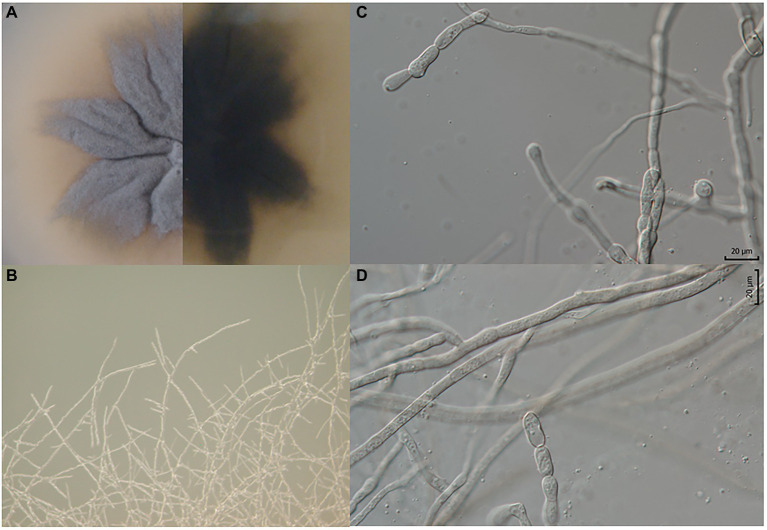
*Pyrenophora gallaeciana*. **(A)** Colony on PDA (front and reverse) (120 h old); **(B)** mycelium on PDA (120 h old); **(C, D)** structures probably involved in asexual reproduction.

***Pyrenophora gallaeciana* J. Poveda, V. M. Rodríguez and P. Velasco, *sp. nov.*** MycoBank MB- 842942. Figure 3 .

*Etymology: gallaeciana* [gal·lækiana]: pertaining to Gallaecia (Galicia), the northwestern region of Spain, where the representatives of this species were isolated.

*Typus:* Spain, Pontevedra, Misión Biológica de Galicia, from roots of *B. oleracea* var. *acephala*, May 2016, J. Poveda, V. M. Rodríguez, and P. Velasco (holotype: CECT-21208, culture ex-type CECT-21208 = CCF 6607 = MB842942).

*Colony diam, 25°C, 7 d (mm):* PDA: 45.3.

*Culture characteristics, 25°C, 7 d:* PDA: The isolate produced dim gray (6D6C72) upper and raisin black (27292B) bottom colonies. The colony morphology is characterized by irregular margins, a wrinkled form, and crateriform elevation (Figure 3A).

*Micromorphology:* Branched hyphae 2.5 μm thick (min.: 2 μm; max.: 3.5 μm) (Figures 3B,C). Constricted hyphae, structures (25–35 × 6–10 μm) probably involved in asexual reproduction, possibly conidia, but with different (much simpler) morphology in comparison to other *Pyrenophora* species (Figures 3C,D).

### Analysis of PGP and enzymatic traits

The potential of the endophytic fungi to promote plant growth and their ability to hydrolyze plant cell compounds (first step to enter the plant tissues) were analyzed through *in vitro* tests. The results are summarized in Table 1. The four endophytic fungal isolates could produce IAA-like molecules at different levels. *A. vagum* did not demonstrate any other PGP mechanism. *S. terrestris* had the ability to solubilize phosphorus and engage in xylanase and amylase activity. *P. gallaeciana* and *F. oxysporum* could produce siderophores and, also, engaged in cellulase and xylanase activity. Furthermore, *P. gallaeciana* exhibited amylase activity.

**Table 1:** PGP attributes of the fungal isolates.

Isolate	IAA-like molecules (μg·mL^−1^)	Siderophores	P solubilization	K solubilization	Cellulase	Xylanase	Amylase
*A. vagum*	9.64	w	−	−	w	w	−
*S. terrestris*	5.75	w	+	−	w	+	+
*P. gallaeciana*	14.67	+	−	−	+	+	+
*F. oxysporum*	29.26	+	−	−	+	+	w

w: weak. Supplementary Figure S5 shows examples of the results of this table.

### Growth promotion In *Brassica* U’s triangle crops

**Figure 4:**
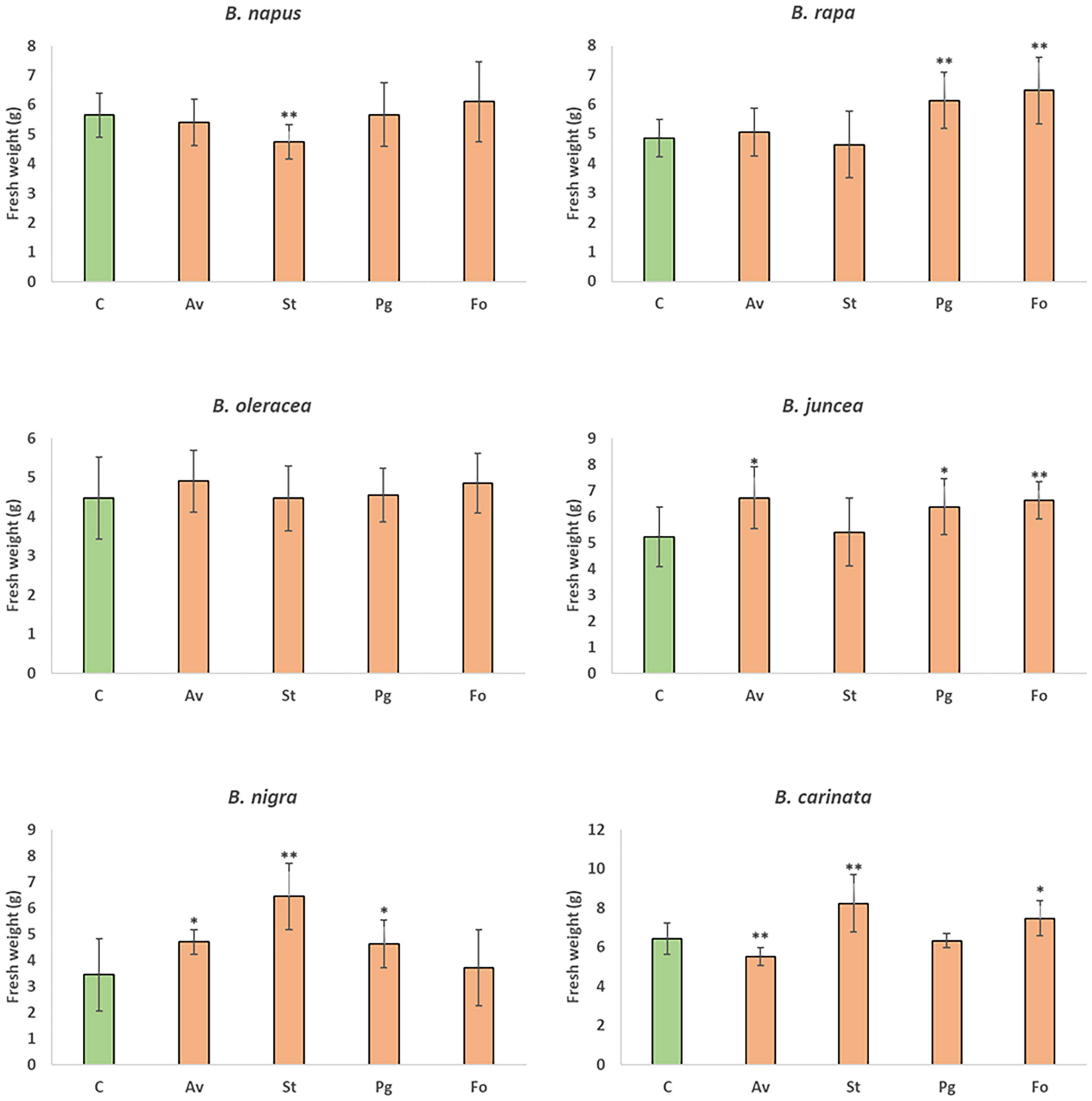
Mean of fresh weights of *B. napus*, *B. rapa*, *B. oleracea* var. *acephala*, *B. juncea*, *B. nigra*, and *B. carinata* plants grown in greenhouse. Plants without inoculation (C) and those inoculated with *A. vagum* (Av), *S. terrestris* (St), *P. gallaeciana* (Pg), and *F. oxysporum* (Fo) were gathered at 12 weeks old. The fresh weights of the aerial parts of 10 plants for treatment were measured and are shown as the mean ± standard deviation. Differences between treatments were calculated using Student’s *t*-test. Asterisks denote significant differences at *p* ≤ 0.05 (*) and *p* ≤ 0.01 (**).

The four endophytic fungi under study were used to evaluate the growth of the six *Brassica* species that make up the U’s triangle. Root inoculation with the endophytic fungus *A. vagum* caused an increase in the weight of the aerial part of *B. juncea* and *B. nigra* compared to uninoculated plants, and significantly reduced the fresh weight of *B. carinata*. In the case of *S. terrestris*, a significant increase in fresh weight was reported in *B. nigra* and *B. carinata* compared to uninoculated plants, and a significant reduction in *B. napus*. The presence of *P. gallaeciana* in roots of *B. rapa*, *B. juncea,* and *B. nigra* significantly increased the fresh weight of the aerial part compared to the plants without fungal inoculation. Finally, root inoculation with *F. oxysporum* produced an increase in the fresh weights of *B. rapa*, *B. juncea,* and *B. carinata* compared to uninoculated plants (Figure 4). The same biomass trend was reported by analysing dry weight (Supplementary Figure S6).

### Root colonization assays with endophytic and pathogenic fungi

To study the role of GSLs in the colonization of *Brassica* roots by pathogenic and endophytic fungi, kale plants were inoculated with the four endophytic fungal isolates (*A. vagum*, *S. terrestris*, *P. gallaeciana,* and *F. oxysporum*) and two pathogens (*L. maculans* and *S. sclerotiorum*) separately.

**Figure 5:**
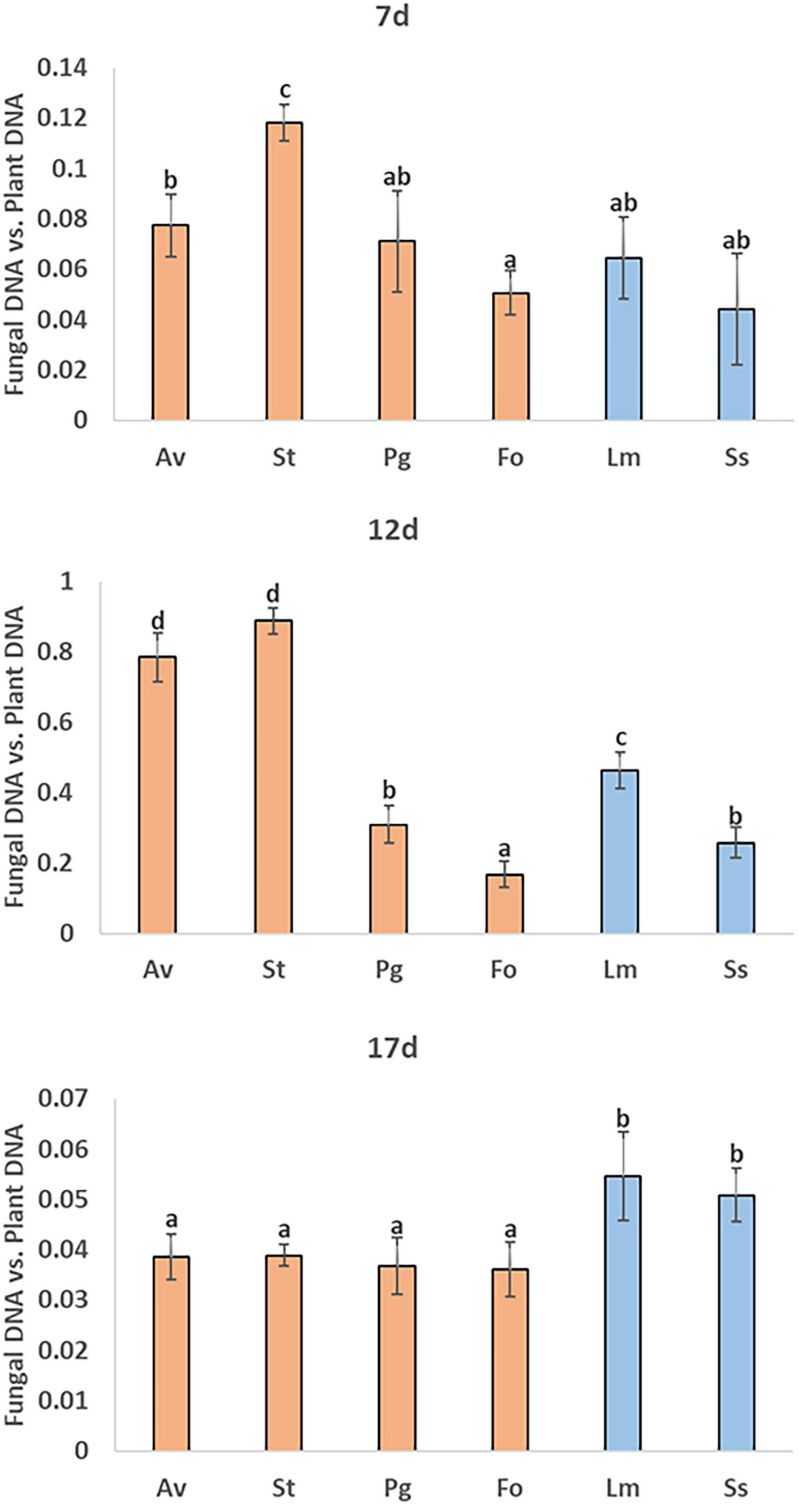
Measurements of kale root colonization by different endophytic and pathogenic fungi using qPCR. *A. vagum* (Av), *S. terrestris* (St), *P. gallaeciana* (Pg), and *F. oxysporum* (Fo) inoculated roots were collected 7-, 12-, and 17-days post-inoculation. Values are the proportion of fungal DNA to plant DNA; means of three root pools (with three plants each) are given with the corresponding standard deviations. One-way analysis of variance (ANOVA) was performed, followed by the Tukey’s test. Different letters represent significant differences (*p* < 0.05).

In the roots collected 7 days post-inoculation, *F. oxysporum* fungus colonized the roots least significantly. Meanwhile, *A. vagum* and *S. terrestris* colonized kale roots significantly more (Figure 5). Both fungi continued to colonize the roots with the greatest significance 12 days post-inoculation, followed in order of significance by *L. maculans*, *P. gallaeciana,* and *S. sclerotiorum*. The fungus that colonized kale roots to a lesser extent continued to be *F. oxysporum* (Figure 5). Finally, 17 days post-inoculation, the four endophytic fungi (*A. vagum*, *S. terrestris*, *P. gallaeciana,* and *F. oxysporum*) colonized the roots without significant differences between them. The two pathogenic fungi (*L. maculans* and *S. sclerotiorum*) showed significantly higher kale root colonization (Figure 5).

#### GSL profiles

**Figure 6:**
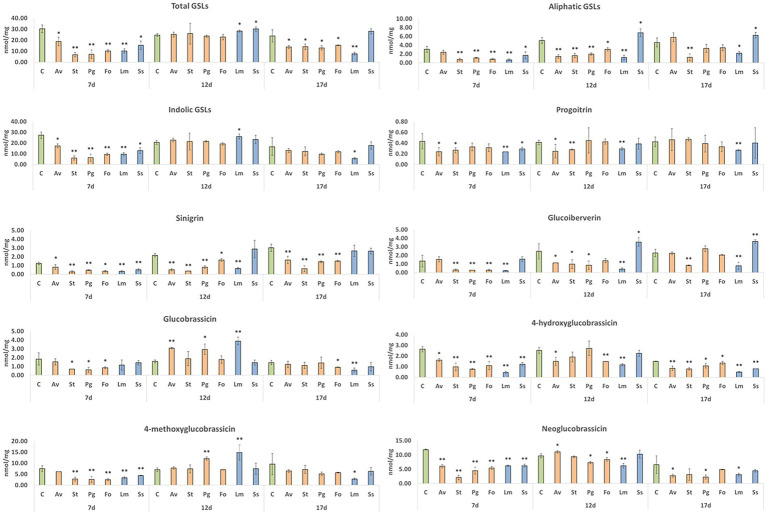
GSL content in kale roots colonized by different endophytic and pathogenic fungi. *A. vagum* (Av), *S. terrestris* (St), *P. gallaeciana* (Pg), and *F. oxysporum* (Fo) inoculated roots were collected 7-, 12-, and 17-days post-inoculation. Data are the means of three root pools (with three plants each) per inoculation with the corresponding standard deviations. Student’s t-test was performed. Asterisks denote significant differences at *p* ≤ 0.05 (*) and *p* ≤ 0.01 (**).

At early stages of inoculation (7 days post-inoculation), fungus colonization significantly reduced the levels of root GSLs. This reduction affected aliphatic and indolic GSLs equally in all tested fungi (Figure 6). However, at 12 days post-inoculation, plants inoculated with endophytic fungi recovered the control amount of GSLs, mainly due to a higher quantity of indolic GSLs, since aliphatic GSLs remained at significantly lower levels that those observed with the control. Roots inoculated with pathogenic fungi accumulated higher levels of total GSLs; there was no common pattern between the two fungi since this increase in plants inoculated with *S. sclerotiorum* was due to the accumulation of aliphatic GSLs, whereas plants inoculated with *L. maculans* accumulated significantly more indolic GSLs. Surprisingly, at 17 days post-inoculation, a significant decrease in the total GSL content was observed, except for in plants inoculated with *S. sclerotiorum*. Quantified aliphatic GSLs included progoitrin, sinigrin, and glucoiberverin. In the case of progoitrin, a significant decrease in its 7 day post-inoculation content was quantified in roots inoculated with *A. vagum*, *S. terrestris*, *L. maculans,* or *S. sclerotiorum*, in comparison with uninoculated plants. The results 12 days post-inoculation continued to be significantly lower in progoitrin in inoculations with *A. vagum*, *S. terrestris*, or *L. maculans*. Meanwhile, 17 days post-inoculation, only the roots inoculated with *L. maculans* showed significant lower levels compared to uninoculated plants (Figure 6). With respect to sinigrin, root inoculation with any fungus caused a significant decrease in kale roots’ sinigrin content compared to uninoculated plants 7 days post-inoculation. The same significantly lower levels were quantified 12 days post-inoculation and 17 days post-inoculation with all fungi, except for *S. sclerotiorum* (at both times) and *L. maculans* (at 17 days post-inoculation) (Figure 6). Finally, in the case of glucoiberverin, in the roots inoculated with fungi *S. terrestris*, *P. gallaeciana*, *F. oxysporum,* or *L. maculans* had significantly lower content glucoiberverin contents 7 days post-inoculation compared to uninoculated plants. Then, 12 days post-inoculation, roots inoculated with *A. vagum*, *S. terrestris*, *P. gallaeciana* or *L. maculans* had significantly lower levels of glucoiberverin than uninoculated plants. However, roots inoculated with *S. sclerotiorum* had significantly higher GSL levels. These significantly higher levels were also quantified in *S. sclerotiorum* 17-days-post-inoculation. In the cases of *S. terrestris* and *L. maculans*, significantly lower levels were maintained than in the uninoculated plants (Figure 6).

As far as indolic GSLs are concerned, glucobrassicin, 4-hydroxyglucobrassicin, 4-methoxyglucobrassicin, and neoglucobrassicin were quantified. The glucobrassicin content decreased significantly in roots inoculated with *S. terrestris*, *P. gallaeciana,* or *F. oxysporum* compared to the uninoculated plants at 7 days post-inoculation. However, 12 days post-inoculation, a significant increase was reported in roots inoculated with *A. vagum*, *P. gallaeciana,* or *L. maculans*. Seventeen days post-inoculation, a significant reduction in the root contents of glucobrassicin was quantified with the fungi *F. oxysporum* and *L. maculans* (Figure 6). In the case of 4-hydroxyglucobrassicin, both 7 and 17 days post-inoculation, the root contents were significantly reduced with the inoculation of all fungi compared to the uninoculated plants. However, 12 days post-inoculation, only the 4-hydroxyglucobrassicin content was significantly reduced with the fungi *A. vagum*, *F. oxysporum*, and *L. maculans* (Figure 6). Seven days post-inoculation, a reduction in the root 4-methoxyglucobrassicin contents of kale plants was quantified with all fungal inoculations, except for *A. vagum*, compared to uninoculated plants. In contrast, 12 days post-inoculation, there was a significant increase in roots inoculated with *P. gallaeciana* and *L. maculans* compared to uninoculated plants. At 17 days post-inoculation, a significant reduction in root inoculation with *L. maculans* was only found (Figure 6). Finally, in the case of neoglucobrassicin, significantly lower levels were reported in roots inoculated with all fungi compared to uninoculated plants at 7 days post-inoculation. However, 12 days post-inoculation, root inoculated with *A. vagum*, *P. gallaeciana*, *F. oxysporum,* or *L. maculans* showed significantly higher neoglucobrassicin contents than uninoculated plants. Finally, the plants inoculated with *A. vagum*, *P. gallaeciana*, or *L. maculans* demonstrated significantly lower levels compared to uninoculated plants at 17 days post-inoculation (Figure 6).

## Discussion

Endophytic fungi have received increasing interest in recent years. However, the numbers of isolates and newly described fungi are still low, especially within *Brassica* crops (Poveda et al., 2022). The ability of different endophytic fungi to promote plant growth in crops, thanks to various mechanisms, has been widely reviewed in recent years (Rana et al., 2019; Rigobelo and Baron, 2021). Although some microbes are adapted to an endophytic lifestyle, to reach roots’ inner tissues, they must first compete and succeed in the rhizosphere, where they can benefit the plant health by providing nutrients and hormones (Jiménez-Gómez et al., 2020). It is important to note that the plant growth-promoting ability may be fungal strain- and plant genotype-specific (Rigobelo and Baron, 2021; Poveda et al., 2021a).

In this study, the specific taxonomic classification of four species of endophytic fungi from kale (*B. oleracea* var. *acephala*) was carried out, with one of them described as a new species. These fungal isolates were obtained in a previous work, although only identified to order or genus level (Poveda et al., 2020a). The four species of endophytic fungi taxonomically described in this work represent the first description, at the species level, of endophytic fungi from kale. These results highlight the importance of studying the diversity of endophytic fungi present in *Brassica* crops.

Here, the production of auxins at different levels was a common PGP trait among the isolates. Although it is known that some strains of *A. vagum* can increase auxin synthesis in plant hosts (Liu and Wei, 2019), this was the first description of the fungus as a putative producer of IAA-like compounds. This mechanism could be involved in its ability to promote the growth of the *Brassica* U triangle crops *B. juncea* and *B. nigra*, the growth promotion previously reported in kale (Poveda et al., 2021a), or *Medicago sativa* growth (Hou et al., 2019). However, in this work the root inoculation of *B. carinata* with *A. vagum* causes a reduction in plant growth, an aspect not previously mentioned for any crop.

The ability of *F. oxysporum* to produce auxins has been previously described, along with its role in promoting the growth of maize (Mehmood et al., 2018). However, our *F. oxysporum* isolate represents the first description of an endophytic isolate of this species as a producer of siderophores. Cellulolytic and xylanase activity have been previously reported for endophyte *F. oxysporum* (Onofre et al., 2013; Farouk et al., 2020). All of these mechanisms could be involved in the growth promotion reported in *B. juncea, B. rapa,* and *B. carinata*, as was previously verified with kale (Poveda et al., 2021a).

The *S. terrestris* isolate solubilizes phosphorus, and it carries out xylanase and amylase activity; ours is the first description of a member of the genus *Setophoma* showing these capacities. Although *Setophoma* species have been previously described as endophytes of Brassicaceae plants, such as horseradish (*Armoracia rusticana*; Szűcs et al., 2018) or kale (Poveda et al., 2021a), this study presents the first description of plant growth-promotion activity by a *Setophoma* species, particularly in *B. nigra* and *B. carinata*. However, our results showed growth reduction when *B. napus* roots were inoculated. A previous study reported the first case of pink root rot in *B. napus* produced by *S. terrestris* (Yang et al., 2017). The thin line between beneficial and pathogenic behavior of some endophytic fungi, when interacting with different hosts, needs further research.

In this work, a new species of endophytic fungi is described: *P. gallaeciana* (meaning: from Galicia, Spain). *P. gallaeciana*-type isolates could produce auxins and siderophores and carry out cellulase, xylanase, and amylase activities. Members of the genus *Pyrenophora*, such as the barley fungal pathogens *P. teres* f. sp. *teres* and *P. graminea* (Ellwood et al., 2010; Bakri et al., 2011; Ismail et al., 2014), showed similar mechanisms. Here, *P. gallaeciana* is a plant growth promoter in *B. juncea, B. rapa,* and *B. nigra*, which is consistent with previous results on the *Pyrenophora*-kale interaction (Poveda et al., 2021a).

The PGP results obtained with these fungal isolates also report important differences according to the mode of application. Poveda et al. (2020a) obtained a higher kale plants growth with inoculation with beet pulp colonized by *F. oxysporum* or *P. gallaeciana* (then identified as *Fusarium* sp. and Pleosporales sp. B, respectively). However, in our current work, inoculation with spores of both fungi did not report a PGP. It is noteworthy that the application of spores allows knowing the exact amount of inoculated endophyte fungus, while the use of mycelium colonizing beet pulp does not, making it impossible to make a real comparison between different fungi. These differences were also reported with the isolate *Trichoderma hamatum*, which did not report PGP capacity in kale plants in its application as mycelium in beet pulp (Poveda et al., 2020c), but showed PGP when applied as spores (Velasco et al., 2021).

The Brassicaceae plants’ pathogenic fungi need to tolerate or eliminate GSLs from plant tissues to colonize and infect them. *Botrytis cinerea* tolerates GSLs through the *mfsG* membrane transporter, which rapidly removes the products of GSLs’ hydrolysis (Vela-Corcía et al., 2019). *S. sclerotiorum* acts directly on these toxic hydrolysis products through an isothiocyanate hydrolase (Chen et al., 2020). In the specific case of endophytic fungi, GSLs’ tolerance that supports them to colonize their hosts’ tissues is not fully understood (Poveda et al., 2022). In the case of *Trichoderma*, the production of myrosinase-binding proteins modifies the hydrolysis of GSLs to allow/contribute to Brassicaceae root colonization (Poveda et al, 2019), with the indole GSLs involved in controling the level of colonization (Poveda, 2021). Other endophytic fungi, including *F. oxysporum* and *S. terrestris*, directly degrade the GSLs present in their hosts and use them as nutrients (Szűcs et al., 2018).

*Brassica*s are non-mycorrhizal plants, with the presence of GSLs precisely proposed as the reason why these plants cannot be mycorrhized (Vierheilig et al., 2000; Anthony et al., 2020). These plants have been described as the main metabolites involved in the control of the endophytic fungal microbiota on this plant family (Plaszkó et al., 2022). Therefore, root colonization by endophytic fungi in *Brassica* plants must be closely related to the GSL content and profile of plant tissues.

The analyses of root colonization levels at 12 days post-inoculation revealed no differences between mock-inoculated plants and endophytic fungi-inoculated ones. Conversely, pathogenic fungi-inoculated plants showed an increased level of colonization. Specifically, with *S. sclerotiorum*, aliphatic GSLs increased. This was previously described in *A. thaliana* infection, where *S. sclerotiorum* induces the expression of synthesis genes (Stotz et al., 2011). Meanwhile, with *L. maculans,* indole GSLs increased, something previously observed in *B. rapa* (Abdel-Farid et al., 2010).

Two common aspects were found as corelated in all endophytic fungal strains vs. tested pathogenic fungal strains at 17 days post-inoculation: root colonization and the sinigrin content. The sinigrin contents were similar for root colonization with any pathogenic strain. However, the sinigrin contents decreased with endophytes’ root colonization. This is possibly due to sinigrin hydrolysis by endophytic fungi and further fungicide activity of the hydrolysis residuals, such as allyl isothiocyanate (Vandicke et al., 2020). Our results suggest that low sinigrin levels detected in roots inoculated with isolates from *P. gallaeciana* and *F. oxysporum* are directly related to their lower levels of root colonization. However, *P. gallaeciana* kale roots’ colonization reached its maximum at 12 days post-inoculation compared to roots inoculated with *F. oxysporum*. In both cases, the 4-hydroxyglucobrassicin content was lower than in mock-inoculated plants, except for roots inoculated with *P. gallaeciana* at 12 days post-inoculation. Thus, 4-hydroxyglucobrassicin hydrolysis products may inhibit both fungi’s root colonization, at different timepoints depending on the fungal strain.

Kale root colonization by endophytes *A. vagum* and *S. terrestris* was higher than for the other fungi at 7 and 12 days post-inoculation, possibly due to a decrease in progroitrin levels, which does not occur 17 days post-inoculation. Similarly, increased root colonization by *L. maculans* at 12 days post-inoculation could also be related to a decrease in the root progoitrin content. Similar GSL profiles for roots colonized by fungal endophytes and certain fungal pathogens could be related to the hemibiotrophic lifestyle, which involves an early stage of endophytism not present in the necrotrophic lifestyle (Newton et al., 2010). Accordingly, lower levels of progoitrin may be related to greater plant colonization by fungal endophytes and pathogens.

Endophytic fungi could induce the expression of synthesis genes, the activation of plant enzymes that hydrolyze them, or act directly against them (Poveda et al., 2022). In this sense, differences in tolerance and/or degradation of GSLs by endophytic and pathogenic fungi may be explained by specific genomic comparisons. However, the sequences of these species are not yet available. Furthermore, the GSL content in the roots does not depend on localized synthesis but can be transported from other plant organs such as leaves (Witzel et al., 2015). Although our results provide some insights, the mechanisms involved in the changes in GSL levels in *Brassica* roots require further research.

In conclusion, this work presents not only the first description at the species level of endophytic fungi of a *B. oleracea* crop but also a description of a new species: *P. gallaeciana*. The strains displayed plant growth-promotion mechanisms, as well as production of hydrolytic enzymes, and could promote the growth of *B. rapa*, *B. napus*, *B. nigra, B. juncea,* and *B. carinata*. Remarkably, our results strongly suggest that progoitrin levels control fungal root colonization independently of the fungal nature, while the hydrolysis of sinigrin reduces colonization only in the case of endophytic fungi.

## Data availability statement

The datasets presented in this study can be found in online repositories. The names of the repository/repositories and accession number(s) can be found in the article/Supplementary material.

## Author contributions

PV and JP conceived the study. JP drafted the initial manuscript. PV, JP, and VR discussed the results and performed statistical analysis of the data. PV, JP, and FS carried out the fungi identification and phylogenetic analysis. MD-U maintained the fungal collection. ZS-S and EM performed the *in vitro* PGP assays. PV, VR, and MD-U performed the *in planta* PGP assays. JP and VR carried out the root colonization assays. PV and VR analyzed the GSLs profile. All authors contributed to the article and approved the submitted version.

## Funding

This research was financially supported by projects RTI2018-096591-B-I00 34 (MCIU/AEI/FEDER, UE) and IN607A 2021/03 (Xunta de Galicia, Spain).

## Acknowledgments

EM acknowledges an European Union’s Horizon 2020 Marie Sklodowska-Curie Actions (Grant Agreement nº 897795). Thanks to Dra. Alba Boscá from the University of Bristol-University of Salamanca for her advice on the choice of the new scientific name (*Pyrenophora gallaeciana*) based on Latin etymology.

## Conflict of interest

The authors declare that the research was conducted in the absence of any commercial or financial relationships that could be construed as a potential conflict of interest.

## Publisher’s note

All claims expressed in this article are solely those of the authors and do not necessarily represent those of their affiliated organizations, or those of the publisher, the editors and the reviewers. Any product that may be evaluated in this article, or claim that may be made by its manufacturer, is not guaranteed or endorsed by the publisher.

## Footnotes

^1^ https://cactiweb.webs.uvigo.es
