# Supplementary material for: Endophytic fungi from kale (Brassica oleracea var. acephala) modify roots-glucosinolate profile and promote plant growth in cultivated Brassica species. First description of Pyrenophora gallaeciana
Source: Front Microbiol. 2022 Oct 5;13:981507. doi: 10.3389/fmicb.2022.981507 (PMC9580329; doi:10.3389/fmicb.2022.981507)
Supplement: Supplementary file 1 [file Data_Sheet_1.docx]

**Table S1:** Isolates examined in this study and their sequences-GeneBank accession numbers.

| **SPECIES** | **ISOLATION Nº** | **MYCOBANK Nº** | **PUBLIC DEPOSIT Nº** | **GENBANK ACCESSION Nº** | | | |
| --- | --- | --- | --- | --- | --- | --- | --- |
|  |  |  |  | **ITS** | **GADPH** | **LSU** | **RPB2** |
| *Acrocalymma vagum* | H22 | - | - | OL830131 | - | - | - |
| *Setophoma terrestris* | H64 | - | - | OL830128 | - | - | - |
| *Pyrenophora gallaeciana* | H441 | MB-842942 | CECT-21208 | OL853521 | OL828338 | OL828339 | OL828340 |
| *Fusarium oxysporum* | H890 | - | - | OL830130 | - | - | - |

**Table S2:** Primers used in the quantification of fungal root colonization.

| **CODE** | **SEQUENCE (5′-3′)** | **USE** | **REFERENCES** |
| --- | --- | --- | --- |
| GADPH-Boa-F | TCAGTTGTTGACCTCACGGTT | Endogenous kale gene | Sotelo et al. (2016) |
| GADPH-Boa-R | CTGTCACCAACGAAGTCAGT |  |  |
| ACT-Acro-F | AGACCTTCAACGCTCCCGCC | Endogenous *Acrocalymma* gene | Rybak et al. (2017) |
| ACT-Acro-R | TGGCGTGGGGAAGAGCGAAAC |  |  |
| ACT-Set-F | AGACCTTCAACGCTCCCGCC | Endogenous *Setophoma* gene | Rybak et al. (2017) |
| ACT-Set-R | TGGCGTGGGGAAGAGCGAAAC |  |  |
| ACT-Pyr-F | CTACGAGCTTCCCGACGGT | Endogenous *Pyrenophora* gene | Amaike et al. (2008) |
| ACT-Pyr-R | TCTGGAGCACGGAAACGC |  |  |
| ACT-Fus-F | CACCACCTTCAACTCCATCA | Endogenous *Fusarium* gene | Hu et al. (2015) |
| ACT-Fus-R | TCGGAGAGACCAGGGTACAT |  |  |
| ACT-Lep-F | AGTGCGATGTCGATGTCAG | Endogenous *Leptosphaeria* gene | Petit‐Houdenot et al. (2019) |
| ACT-Lep-R | AAGAGCGGTGATTTCCTTCT |  |  |
| ACT-Scl-F | AAGCCGTCCTCTCCCTTTAC | Endogenous *Sclerotinia* gene | Maximiano et al. (2020) |
| ACT-Scl-R | ATGGCGTGAGGAAGTGAGAA |  |  |
