# Supplementary figures and images for: Endophytic fungi from kale (Brassica oleracea var. acephala) modify roots-glucosinolate profile and promote plant growth in cultivated Brassica species. First description of Pyrenophora gallaeciana
Source: Front Microbiol. 2022 Oct 5;13:981507. doi: 10.3389/fmicb.2022.981507 (PMC9580329; doi:10.3389/fmicb.2022.981507)

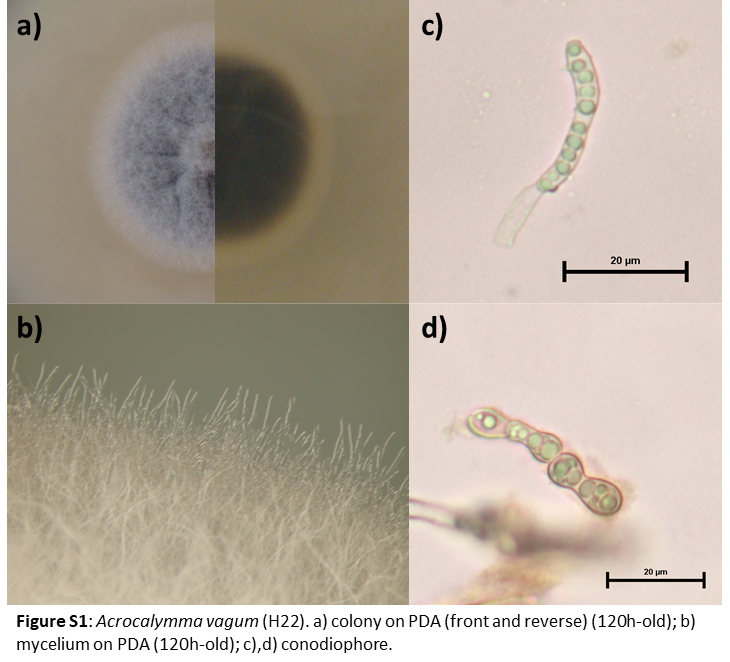

Supplement: Supplementary file 2 [file Image_1.TIF]

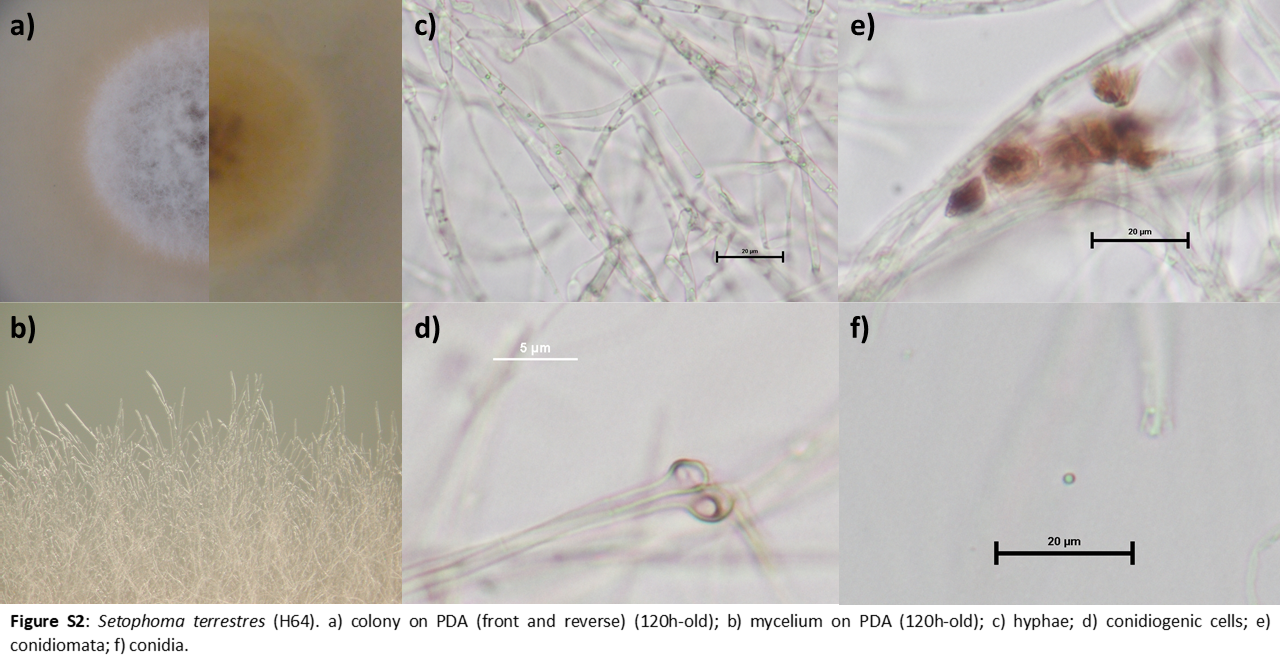

Supplement: Supplementary file 3 [file Image_2.TIF]

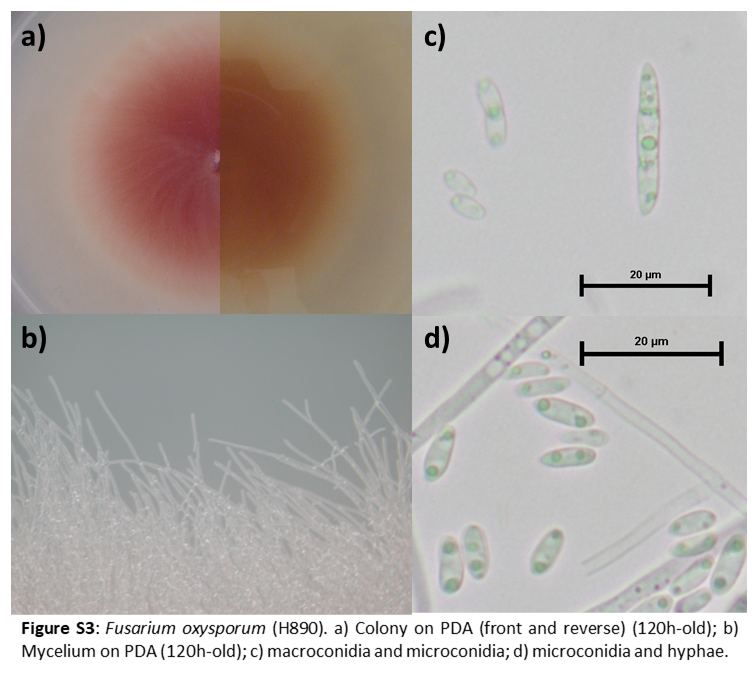

Supplement: Supplementary file 4 [file Image_3.TIF]

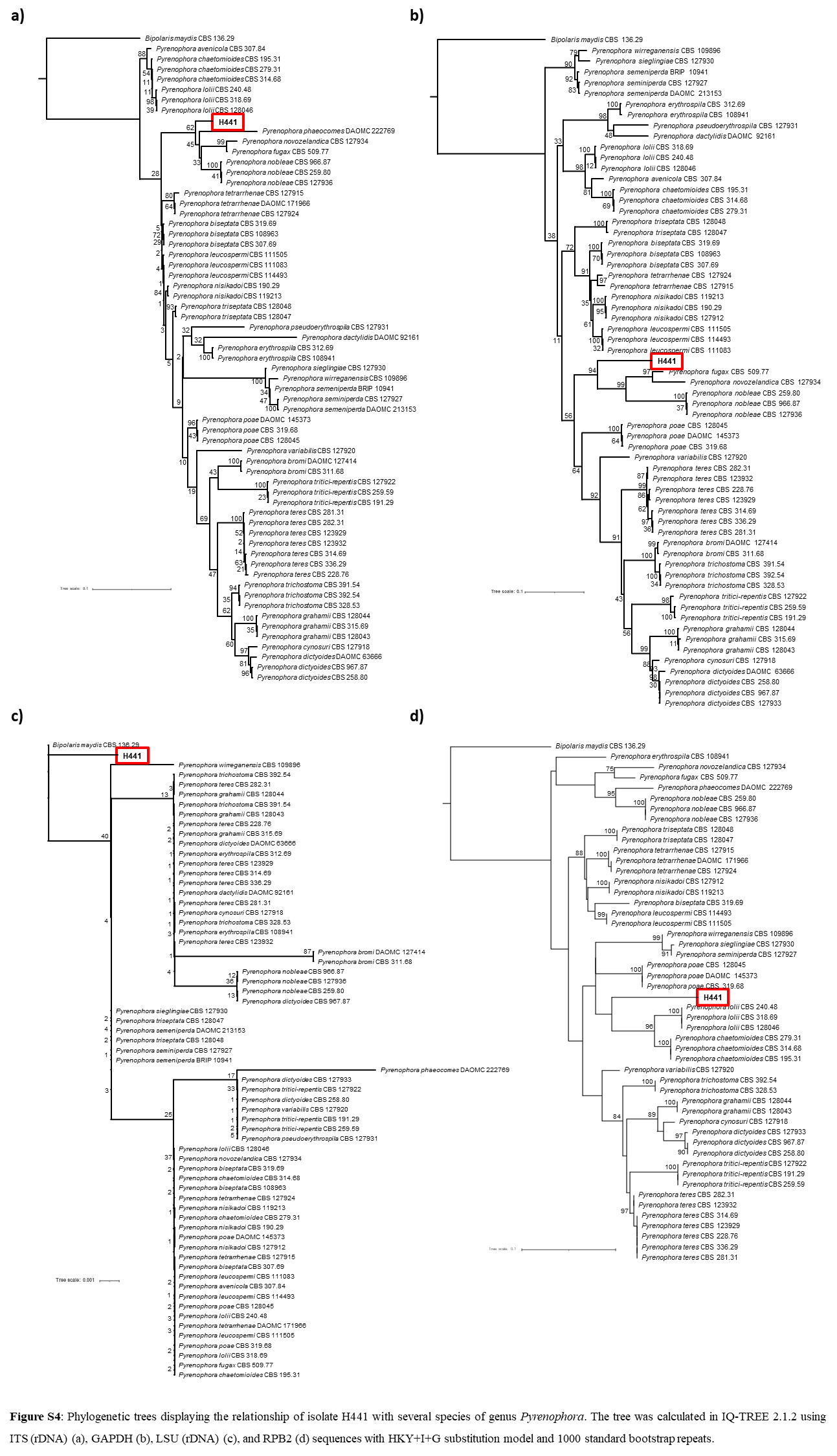

Supplement: Supplementary file 5 [file Image_4.TIF]

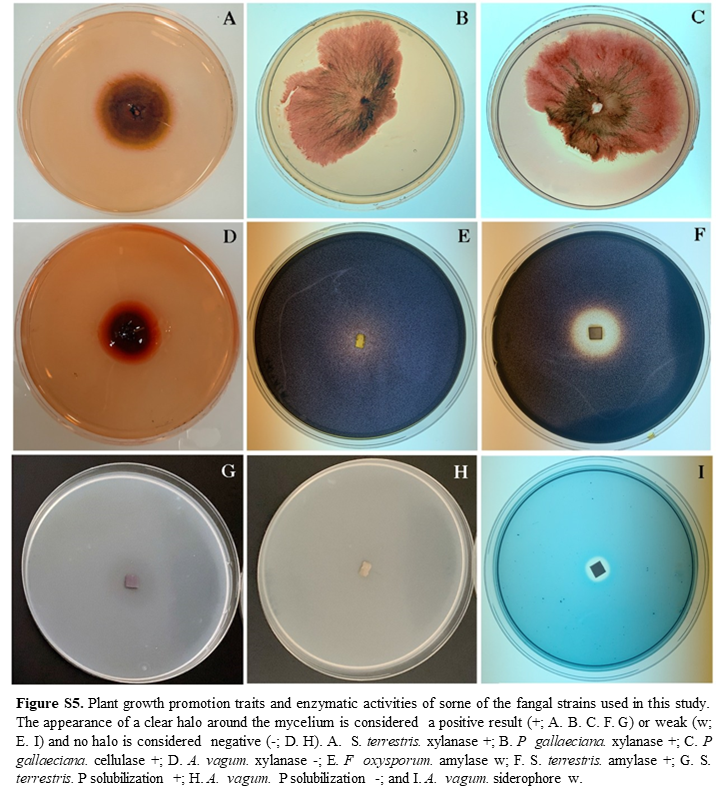

Supplement: Supplementary file 6 [file Image_5.TIF]
